# Influence of Brewing Methods on the Bioactive and Mineral Composition of Coffee Beverages

**DOI:** 10.3390/molecules30204080

**Published:** 2025-10-14

**Authors:** Monika Sijko-Szpańska, Iwona Mystkowska, Aleksandra Dmitrowicz

**Affiliations:** 1 Regional Research Centre on Environment, Agriculture and Innovative Technologies EKO-AGRO-TECH, John Paul II University in Biala Podlaska, Sidorska 95/97, 21-500 Biala Podlaska, Poland; m.sijko-szpanska@akademiabialska.pl; 2 Department of Dietetics, John Paul II University in Biala Podlaska, Sidorska Street 95/97, 21-500 Biala Podlaska, Poland; i.mystkowska@dyd.akademiabialska.pl

**Keywords:** coffee, caffeine, polyphenol, minerals, HPLC, ICP-OES, brewing

## Abstract

The chemical profile of coffee depends on numerous factors, the complexity of which makes it difficult to clearly assess their influence. The aim of this study was to comprehensively evaluate the impact of selected coffee brewing methods (Espresso, Simple Infusion, French Press, V60), taking into account the coffee species (Arabica, Robusta, Blends), the degree of roasting (light, medium, dark) and the geographical origin (single-origin and multi-origin) on the chemical composition of the brew. Eighteen different types of coffee, which differ in the aforementioned characteristics, were analyzed. The caffeine content (using high-performance liquid chromatography), the total phenolic content (TPC; using a spectrophotometric method), and selected minerals (calcium, iron, potassium, magnesium, sodium, phosphorus, zinc; using Inductively Coupled Plasma–Optical Emission Spectrometry) were analyzed. The analysis showed that both the brewing method and the species had a significant influence on the chemical profile of the resulting brews, while the degree of roasting and the origin showed no significant influence. The Espresso method showed the highest caffeine, TPC, potassium, magnesium, and phosphorus content, the V60 method—calcium, iron, and sodium, and the French Press and Simple Infusion methods showed intermediate values. Robusta coffee contained more caffeine and TPC, Arabica contained more magnesium, and Blend showed medium values for both species. The results obtained may have practical implications for both consumers and the coffee industry, supporting informed decision-making and the refinement of brewing methods.

## 1. Introduction

Coffee is one of the most widely consumed beverages in the world. In addition to its sensory qualities, it is also increasingly the subject of research due to its potentially health-promoting properties. In recent years, numerous scientific studies have focused on analyzing the relationship between coffee consumption and the reduction in the risk of a number of diseases and identifying the compounds responsible for these effects [1,2,3,4,5,6]. However, this issue is very complex, mainly due to the complex chemical profile of coffee, which includes numerous bioactive compounds.

One of the most thoroughly investigated components of coffee is caffeine, the content of which can vary considerably depending on a number of factors, particularly portion size, so that its concentration in the brew has a wide range [7]. However, it is worth noting that this is not the only compound with potential biological effects. The polyphenols contained in coffee, among which chlorogenic acids dominate, are considered to be of great importance. These are esters of caffeic acid, ferulic acid, or p-coumaric acid with quinic acid [8,9,10]. Coffee infusions can also be a source of minerals, including manganese, zinc, magnesium, and potassium [11,12]. However, the content of all components in coffee varies widely, with many variables influencing the final composition of the brew.

The species of coffee is a key factor determining the chemical composition of the brew. Arabica beans, for example, have a lower caffeine content than Robusta beans [13]. Numerous studies confirm that the chemical composition of the coffee brew also depends on factors such as the degree of roasting of the beans or their place of origin [14,15]. It should also be noted that the degree of roasting significantly affects the chemical composition of coffee, as higher roasting temperatures lead to partial degradation of chlorogenic acids and the formation of melanoidins and other Maillard reaction products [14]. The preparation method is also an important factor that modifies the content of bioactive compounds and minerals in the finished beverage. In addition to traditional brewing methods, brewing methods such as French Press and V60 are becoming increasingly popular. These methods are recognized by both consumers and researchers who analyze the chemical composition, sensory profile, and biological activity of brews [16,17,18]. Within the method, there are many determinants such as the ratio of coffee to water, brewing time, water temperature, and pressure used [19,20]. Studies have consistently shown that these parameters can significantly influence the levels of caffeine, polyphenols, lipids, and other bioactive compounds in coffee [16,17,18,19,20]. For example, higher brewing temperatures and finer grinding increase both extraction rate and overall extraction yield, while the coffee-to-water ratio strongly affects extraction yield and titratable acidity but has little impact on extraction rate [20]. Furthermore, the efficiency of immersion-based methods depends on complex interactions among grinding size, water temperature, brewing ratio, and steeping time, highlighting the multidimensional nature of the extraction process [20]. Low-temperature brewing, typical of traditional cold brew, slows down extraction and requires prolonged brewing times, whereas modern approaches such as percolation allow for shorter brewing times while achieving higher levels of bioactive compounds, lipids, and improved sensory properties [17]. Comparing these findings, it is evident that optimizing brewing parameters requires an understanding of how each factor and their interactions modulate both chemical composition and sensory characteristics of coffee.

Due to the high number of raw material and technology variables, determining the content of the individual components remains challenging. Despite the many studies available, there is still a clear research gap in analyzing how these factors affect the chemical profile of coffee. The aim of this study was to comprehensively evaluate the impact of selected brewing methods (Espresso, Simple Infusion, French Press, V60) on the chemical profile of coffee, considering coffee species (Arabica, Robusta, Blend), degrees of roasting (light, medium, dark), and geographical origins (single-origin and multi-origin). The study focused on key compounds, including caffeine, total phenolic content (TPC), and selected minerals: (Ca), potassium (K), magnesium (Mg), sodium (Na), phosphorus (P), iron (Fe), and zinc (Zn). To the best of the authors’ knowledge, this is one of the few wide-ranging studies that simultaneously consider the diversity of raw materials and different brewing methods, thereby enabling a multifaceted characterization of the chemical and functional profile of coffee infusions.

## 2. Results

The content of the analyzed compounds in coffee brews was described regarding the brewing methods and subsequently evaluated in the context of coffee species, roasting degree, and coffee origin.

### 2.1. Caffeine, TPC, and Mineral Content Across Brewing Methods

**Figure 1:**
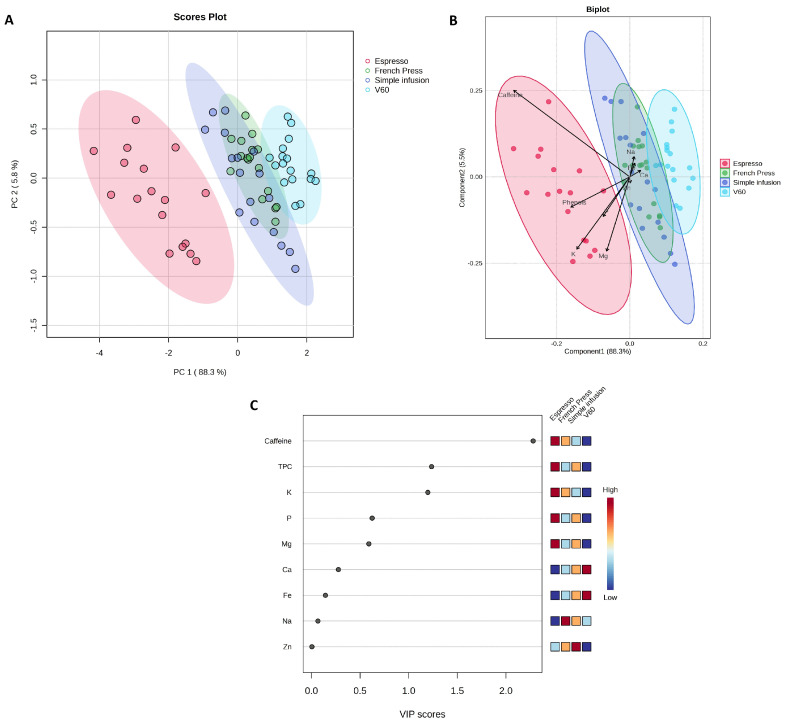
(**A**) Principal Component Analysis (PCA) based on brewing methods (F = 81.016; R^2^ = 0.79156; *p* = 0.001); (**B**) Partial Least Squares–Discriminant Analysis (PLS-DA) scores plotted according to brewing methods (R^2^ = 0.809; Q^2^ = 0.741; cross-validation error rate = 0.147; permutation test *p* < 0.001); (**C**) Variable Importance in Projection (VIP) scores for the different parameters, plotted by brewing method. Figure generated based on analysis performed using the web-based software MetaboAnalyst 6.0 (https://www.metaboanalyst.ca/, accessed on 16 July 2025).

To assess the chemical differences between coffee samples prepared using four different brewing methods (Espresso, French Press, Simple Infusion, and V60), a principal component analysis (PCA) was performed (Figure 1A).

The first two principal components explain 94.1% of the total variation in the data. PCA and PLS-DA suggest tendencies for certain brewing methods to differ (e.g., Espresso), whereas substantial overlap between other methods (e.g., Simple Infusion and French Press) indicates that the differences are not fully discriminative. The Espresso method (red points) appears as a relatively distinct group, while the V60 method shows partial separation from the others. In contrast, the Simple Infusion (purple points) and French Press (green points) methods partially overlap, suggesting similar chemical profiles. A PLS-DA (Figure 1B) was performed to identify the variables responsible for the differences detected between the brewing methods. The results indicated that caffeine content, TPC, K, and Mg had the most significant influence in distinguishing samples prepared with the Espresso method from the others. In contrast, the samples prepared using the other methods (Simple Infusion, French Press, V60) were characterized by higher Na, Ca, and Zn content. The results of Variable Importance Projection (VIP) analysis (Figure 1C) confirmed that caffeine content, TPC, and K are the variables with the highest VIP values (>1), indicating their key role in differentiating the chemical profile of coffees prepared with different brewing methods.

**Figure 2:**
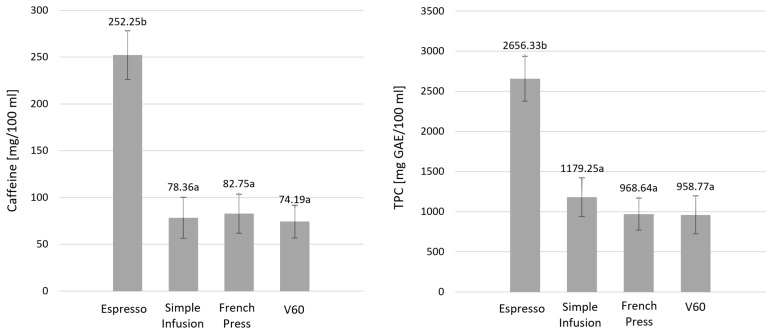
Caffeine and total polyphenol content (TPC) in infusions prepared using different brewing methods. Values are presented as mean ± standard deviation (*n* = 3). Different lowercase letters denote statistically significant differences between brewing methods (*p* < 0.05), as determined by one-way ANOVA followed by Tukey’s post hoc test.

Figure 2 shows the caffeine content and TPC using the brewing method.

The caffeine content of coffee samples prepared by the Espresso method was significantly higher than the other brewing methods, for which caffeine concentrations did not differ considerably (*p* < 0.001). Similar results were obtained against TPC, with the Espresso method showing significantly higher values against the other extraction methods (*p* < 0.001).

Table 1 summarizes the mineral content of the coffee samples according to the brewing method used.

The analysis showed significant differences in the content of all minerals analyzed (except Zn) depending on the brewing method. The Espresso method had the highest content of selected macrominerals—K, Mg, and P—whose concentrations were significantly higher than all other brewing methods. In contrast, brews prepared using the V60 method showed the highest Ca, Fe, and Na content, statistically distinguishing them from the other extraction techniques. The French Press method showed moderate mineral elements extraction efficiency, not reaching the highest values for any elements. However, for selected minerals, it was comparable or slightly lower than the V60 and Espresso methods. The lowest concentrations of almost all minerals analyzed (except Zn) were observed in samples prepared using the Simple Infusion method.

### 2.2. Content of Caffeine, Total Polyphenols, and Mineral Content Across Coffee Species

**Figure 3:**
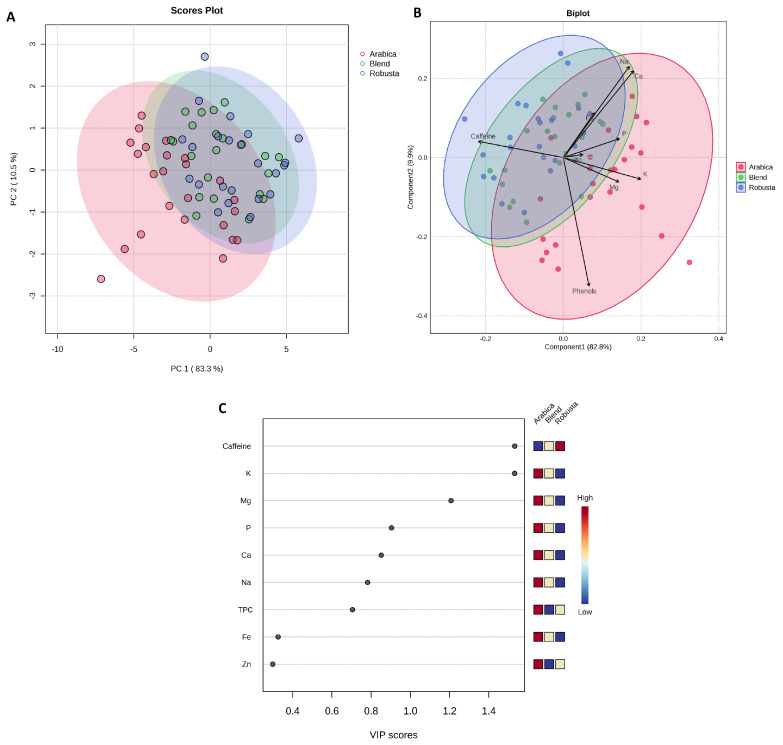
(**A**) Principal Component Analysis (PCA) based on coffee species (F = 15.795; R^2^ = 0.3237; *p* = 0.001); (**B**) Partial Least Squares–Discriminant Analysis (PLS-DA) scores plotted according to coffee species (R^2^ = 0.753; Q^2^ = 0.673; cross-validation error rate = 0.210; permutation test *p* < 0.001); (**C**) Variable Importance in Projection (VIP) scores for the different parameters. Figure generated based on analysis performed using the web-based software MetaboAnalyst 6.0 (https://www.metaboanalyst.ca/ (accessed on 16 July 2025)).

PCA was conducted to examine the variability of samples by coffee species (Arabica, Robusta, Blend), as shown in Figure 3.

The first two components comprise 93.8% of the total variation in the data. A partial overlap of the groups was observed, but the Arabica coffee samples (red points) form a appear as a relatively distinct group from the Robusta coffee samples (blue points), while Blend coffee (green points) occupies an intermediate position. The PLS-DA indicated trends in the chemical profile among the analyzed coffee species. The vectors of the variables suggested that the caffeine content contributes primarily to the differentiation of the Robusta samples, while Arabica shows relatively higher TPC, Mg, and K content. The Blend occupies an intermediate position, reflecting its complex chemical profile. VIP analysis showed that caffeine, K, and Mg reached the highest VIP values (>1), highlighting their role in contributing to the observed differences in the chemical profile of the analyzed coffees.

**Figure 4:**
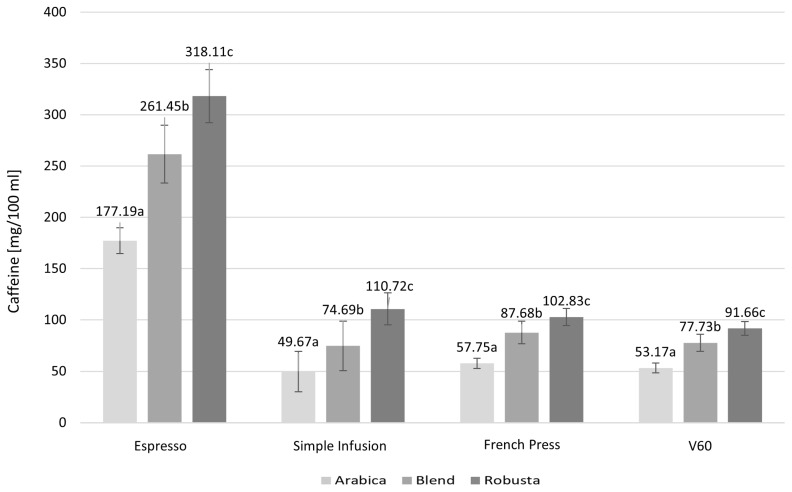
Caffeine content in infusions prepared using different brewing methods and depending on the species. Values are presented as mean ± standard deviation (*n* = 3). Different lowercase letters denote statistically significant differences between brewing methods (*p* < 0.05), as determined by one-way ANOVA followed by Tukey’s post hoc test.

The caffeine content of coffee species and brewing methods is shown in Figure 4.

Coffee made from Robusta beans had the highest caffeine content in each preparation type analyzed, while the lowest caffeine content was recorded in samples prepared from Arabica coffee. Blend samples, a mixture of Arabica and Robusta, had medium caffeine concentrations, suggesting that their chemical profile is proportionally dependent on the proportion of each species in the blend. The differences in caffeine content between brewing methods were statistically significant for each method analyzed: *p* < 0.001 for Espresso, *p* = 0.0004 for Simple Infusion, *p* < 0.001 for French Press, and *p* < 0.001 for the V60 method.

**Figure 5:**
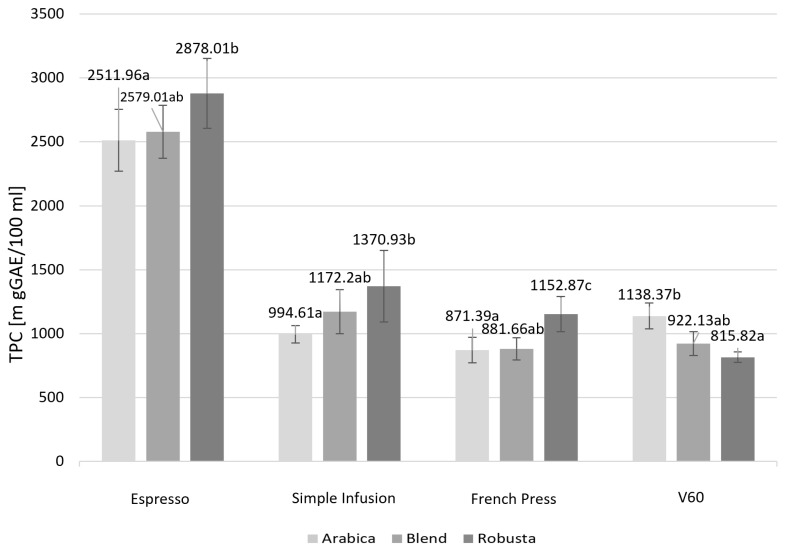
Total polyphenol content (TPC) in infusions prepared using different brewing methods and depending on the species. Values are presented as mean ± standard deviation (*n* = 3). Different lowercase letters denote statistically significant differences between brewing methods (*p* < 0.05), as determined by one-way ANOVA followed by Tukey’s post hoc test.

Figure 5 presents the TPC depending on the coffee species and brewing method.

Significant differences in TPC were observed depending on the brewing method and coffee species. All brewing methods analyzed showed statistically significant differences in TPC between Arabica and Robusta. The Espresso, Simple Infusion, and French Press methods showed the highest TPC values for Robusta, while the V60 method resulted in a higher TPC for Arabica samples. Blend coffee was characterized by a medium TPC, regardless of the brewing method used, except the French Press method, where the TPC was similar to that observed for Arabica. The differences in TPC between coffee species were statistically significant for all brewing methods, with significance levels of *p* = 0.0439 for Espresso, *p* = 0.0338 for Simple Infusion, *p* = 0.0105 for French Press, and *p* = 0.0294 for V60.

The content of most of the analyzed minerals showed no significant differences between the coffee species within each brewing method (Table 2).

The Espresso method showed significant differences in Mg (the highest concentration recorded in Arabica) and Zn, with the highest concentration observed in Arabica and Robusta. The minerals concentrations in the Simple Infusion method were comparable regardless of coffee species. In the French Press method, the highest concentrations of all minerals analyzed were observed in Arabica; in the case of Mg, the difference to the other varieties was significant. With the V60 method, the Fe and Mg contents showed substantial differences. As with the French Press method, the Mg content was highest in Arabica with the V60 method, while the Fe content was lowest in Blend coffee.

### 2.3. Content of Caffeine, Total Polyphenols, and Mineral Content Across Degree of Roasting and Origin

**Figure 6:**
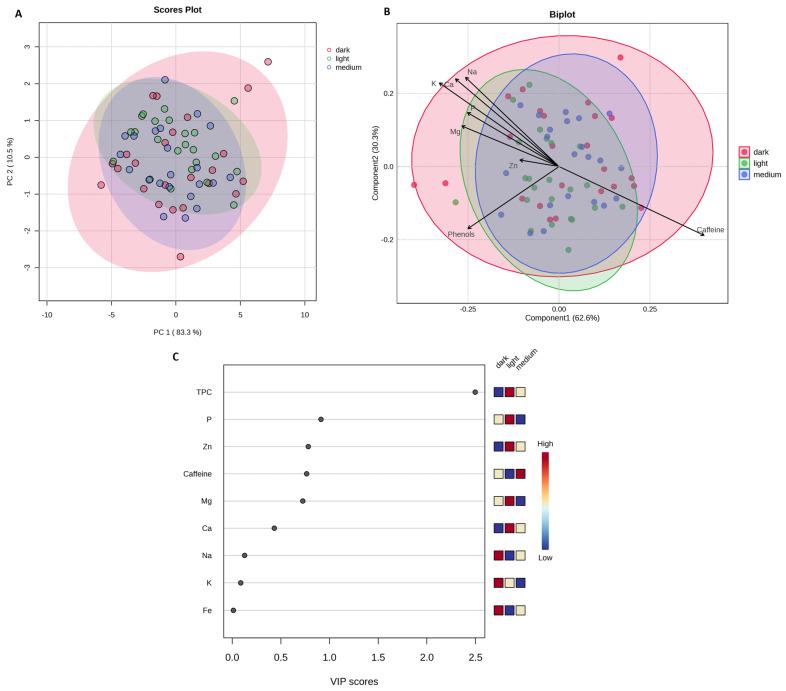
(**A**) Principal Component Analysis (PCA) based on degree of roasting (F = 0.32784; R^2^ = 0.0094; *p* = 0.784); (**B**) Partial Least Squares–Discriminant Analysis (PLS-DA) scores plotted according to degree of roasting (R^2^ = 0.300; Q^2^ = 0.019; cross-validation error rate = 0.659; permutation test *p* = 0.187); (**C**) Variable Importance in Projection (VIP) scores for the different parameters. Figure generated based on analysis performed using the web-based software MetaboAnalyst 6.0 (https://www.metaboanalyst.ca/ (accessed on 16 July 2025)).

**Figure 7:**
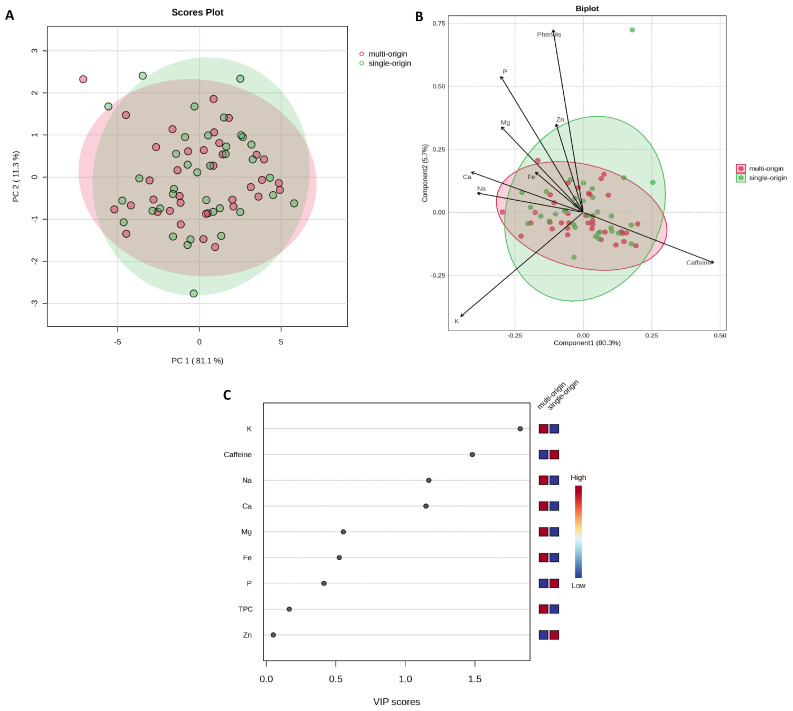
(**A**) Principal Component Analysis (PCA) based on geographical origin (F = 0.0977; R^2^ = 0.0014; *p* = 0.862); (**B**) Partial Least Squares–Discriminant Analysis (PLS-DA) scores plotted according to geographical origin (R^2^ = 0.037; Q^2^ = −0.220; cross-validation error rate = 0.571; permutation test *p* = 0.974); (**C**) Variable Importance in Projection (VIP) scores for the different parameters. Figure generated based on analysis performed using the web-based software MetaboAnalyst 6.0 (https://www.metaboanalyst.ca/ (accessed on 16 July 2025)).

The effects of the roasting degree and the coffee bean origin on caffeine, TPC, and mineral content were also analyzed (Figure 6 and Figure 7).

Both the PCA and PLS-DA analyses showed no clear tendencies for separation of the samples according to the degree of roasting (Figure 6) or the origin of the beans (Figure 7). These results suggest that the analyzed factors had limited influence on the content of the analyzed compounds. The detailed results, including the mean values ± standard deviation and the results of the statistical significance tests, are shown in Tables S1 and S2 in the Supplementary Material.

### 2.4. Brewing Method and Coffee Species as Factors for Differentiating the Chemical Profile of Coffees

**Figure 8:**
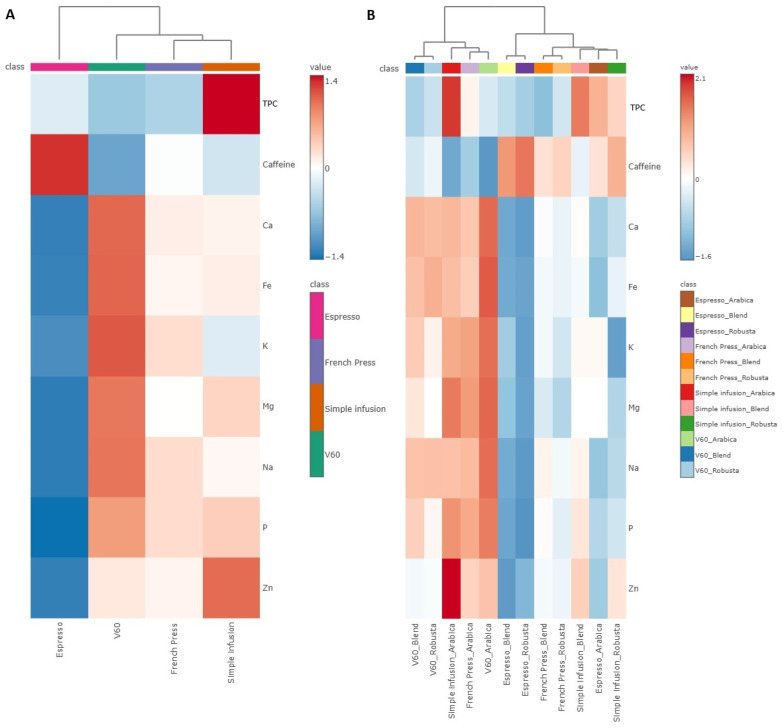
Hierarchical Clustering Heatmaps illustrating the influence of (**A**) brewing method and (**B**) coffee species on the content of selected bioactive compounds and minerals in coffee brews. The heatmaps are based on normalized values. Red indicates higher, and blue lower compound concentrations. Figure generated based on analysis performed using the web-based software MetaboAnalyst 6.0 (https://www.metaboanalyst.ca/ (accessed on 16 July 2025)).

The differences in the content of bioactive compounds and minerals depending on the brewing methods and coffee species, heat maps (Figure 8) are presented to illustrate the concentration patterns for the analyzed parameters. This allows us to identify the content patterns of each chemical parameter depending on the brewing method and coffee species.

Significant Espresso coffee was characterized by the highest caffeine content and the lowest content of analyzed minerals. In contrast, the V60 method was characterised by a high content of most of the minerals analyzed, in particular a high content of K, Ca, Fe, Mg, and Na, but a low content of caffeine. The coffee prepared using the French Press method had medium values for most of the parameters tested and neither the highest nor the lowest concentrations of the minerals analyzed, indicating a moderate extraction of both caffeine and minerals. In contrast, the coffee prepared with the Simple Infusion method had the highest TPC and Zn content, while the caffeine content was relatively low. Since coffee species appeared to have the most pronounced influence on the content of the analyzed components, as suggested by the PCA in which the samples tended to group by type, a more detailed investigation was conducted using a heat map with Hierarchical Clustering, taking this factor into account. Within the Espresso coffee, the Robusta and Blend coffee samples had a higher caffeine content than Arabica. The V60 method was characterized by a higher mineral content, with the highest values recorded in Arabica coffee infusions. In the French method, the content of the analyzed compounds was in the medium range, but after taking into account the coffee species, it is worth noting that Arabica infusions contained higher values of mineral substances, while Robusta and Blend contained more caffeine. In the coffee infusions prepared using the Simple Infusion method, Arabica coffee had a significantly higher content of Zn and other mineral components, while Robusta and Blend coffees were characterized by an increased content of TPC.

### 2.5. Chemical Composition of a Portion of Infused Coffee Depending on Brewing Method and the Percentage of the Daily Requirement Covered

Table 3 shows the content of caffeine, TPC and selected minerals (Ca, Mg, K, Na, P, Fe and Zn) in one serving of coffee infusion prepared by four methods: Espresso (25 mL), Simple Infusion, French Press and V60 (250 mL).

Espresso coffee contained a considerable amount of caffeine despite the smallest serving size, but this was the lowest value per serving among the methods analyzed. The other brewing methods provided similar amounts of caffeine per serving. The highest TPC was recorded in the coffee prepared using the Simple Infusion method and the lowest in the Espresso. In terms of mineral content, the highest concentrations of most minerals were recorded in a serving of coffee prepared using the V60 method, while the lowest concentrations were recorded in Espresso. Methods such as French Press and Simple Infusion had intermediate values, with Simple Infusion having the highest Zn content per serving of coffee.

The percentage realization of the daily requirement for caffeine and selected minerals, calculated on the basis of EFSA’s recommendations [21], is shown in Table 4, assuming the consumption of one serving of coffee prepared by each of the methods analyzed. Values were related to the recommended standards, with values for components whose standards differ by gender presented separately for women and men. In the calculations for ingredients whose norms have a range of values, the lower value was taken.

Although Espresso has a relatively small volume, it delivers a notable amount of caffeine (more than 15% for the EFSA-recognized safe daily intake), while the pour-over methods (Simple Infusion, French Press and V60) showed significantly higher values, covering about half of that. Brews prepared with pour-over methods provided significantly higher amounts of minerals compared to Espresso. In the case of minerals, coffee’s contribution to covering the daily requirement is marginal—most often less than 2.5%. The highest percentages of recommended daily intake were recorded for Ca (1.72%), Mg (up to 2.36%), K (1.54%), Na (0.75%), P (1.90%) and Fe (up to 6.18%), with the highest contributions for most of these components observed in brews prepared using the V60 method. Although the brew obtained with this method is relatively mild, it demonstrates the greatest capacity for supplying mineral elements, especially Fe. These values apply to one serving of brew, if two servings were consumed, coffee’s contribution to minerals requirements would increase significantly. For example, the Fe content from two servings of coffee prepared using the V60 method would already correspond to nearly 15% of the daily requirement in men. However, for the other minerals, even with such consumption, the contribution would not exceed 10%.

## 3. Discussion

To the best of our knowledge, this is one of the few comprehensive studies of its kind to consider a wide range of variables affecting the chemical profile of coffee brews. The study evaluated the impact of selected brewing methods, allowing for a broader approach than previous publications, which typically analyzed only one method. In addition, a variety of coffees were considered, rather than just one type of raw material or one degree of roasting, as was the case in most previous studies. Coffees of different species (Arabica, Robusta, blends), with different degrees of roasting (light, medium, dark) and geographical origin (single and multi-origin) were analyzed. A number of chemical parameters were evaluated, including caffeine content, TPC, and selected minerals, which allowed for the analysis of the chemical profile of different coffees from several points of view.

Regarding the first parameter analyzed, caffeine, the study results showed that both the brewing method and the coffee species tended to influence caffeine content. Among the analyzed brewing methods, Espresso coffee had the highest caffeine content per 100 mL; however, considering typical serving sizes (25–30 mL for Espresso and 200–250 mL for other methods), the total caffeine intake per serving may not be higher than that of other brewing methods. This result is supported by the PLS-DA analysis, in which the caffeine content was one of the key variables contributing to the differentiation of Espresso samples from the other methods. The Espresso method is an intensive extraction method in which high pressure is applied with a short contact time with water, which, together with high temperature, contributes to an efficient release of caffeine [22]. Similar results can be found in the literature [23]. Espresso preparation inherently involves a slightly broader temperature range (90–96 °C), which, together with pressure, may influence extraction kinetics compared to other methods (92–93 °C). In other methods, such as French Press, Simple Infusion, and V60, the caffeine content was lower than in Espresso and did not differ significantly, suggesting a comparable efficiency of caffeine extraction. The methods used the same amount of coffee and water, similar water temperatures (92 or 93 °C), and similar brewing times (5 min for French Press and Simple Infusion; 3.5 min for V60). Although temperature differences can influence the kinetics of caffeine extraction, all brewing procedures were performed under controlled conditions, with water temperature monitored in the kettle to ensure reproducibility. The slightly lower caffeine content in the V60 method, although statistically insignificant, could be due to the shorter extraction time and the faster flow of water through the paper filter. In a study by Angeloni et al. [23], the caffeine content of coffee prepared using the V60 and French Press methods was also comparable. In contrast, another study found differences in caffeine content between these brewing methods, but the relationship was only apparent in light- and dark-roasted coffees; the caffeine content was similar in medium-roasted samples [18]. On the other hand, Muzykiewicz et al. reported that factors such as brewing time (9 h vs. 24 h, as well as 4 min vs. 10 min) and water temperature (below and above 90 °C) had a smaller impact on the analyzed properties of coffee infusions, suggesting that under certain conditions these parameters may be less decisive for caffeine levels than the choice of brewing method itself [19]. These contradictory findings indicate that the influence of temperature and brewing time may depend on additional factors such as coffee matrix, roast degree, or extraction dynamics specific to the method, underlining the complexity of interpreting results across different studies.

However, when analyzing the results by species, Robusta coffee contained a higher caffeine content than Arabica coffee, regardless of the brewing method used. Coffee Blends containing a mixture of both species had medium values. The results are consistent with literature data [24]. Similar correlations were also observed in studies by other authors, both for Espresso coffee [25,26] and for coffee prepared using the Moka method [26] or by adding cold water and boiling [27].

Several studies have confirmed that the caffeine content remains relatively constant with roasting temperature, suggesting that caffeine is thermostable [28,29,30,31]. Most of the studies involved Arabica coffee (with the exception of the study by Tfouni et al. [27], which also included Robusta beans), and the maximum temperature was 220 °C. These studies also used different roasting times (6 to 30 min), suggesting that time and temperature do not significantly affect caffeine degradation. Our study also found no significant differences in caffeine content between samples with different degrees of roasting. Individual reports suggest that a higher degree of roasting may result in a lower caffeine content, e.g., in a study by Górecki and Hallmann [32] who used a temperature of 186.5 °C but different roasting times (7, 8 and 14 min), but these results do not appear to be consistent with the majority of the literature and this requires further analysis.

Some studies have suggested that the origin of the coffee may influence the caffeine content of the coffee [33,34]. Regarding the origin of the beans declared by the manufacturers, it is possible that this information was not always accurate or factually correct, which may have influenced our study’s lack of clear associations between origin and caffeine content.

The brewing method and the species of coffee also had a significant influence on the TPC of the prepared brews. In our study, the Espresso method had the highest TPC of all brewing methods investigated, which is consistent with the observations of Ludwig et al. [35], who showed that high pressure is a key factor for increasing the efficiency of polyphenol extraction in this method. In contrast, the lower TPC observed in the other methods could be attributed to factors such as the lower fineness of the coffee grounds and the longer extraction time, while oxidative degradation of phenolic compounds may be limited due to predominantly anaerobic brewing conditions. Similar observations regarding the effect of the brewing method on the content of phenolic compounds are confirmed by the results of different studies. Derossi et al. [36] reported that Espresso coffee had the highest TPC, reaching 2201 mg GAE/100 mL, a value consistent with the results of the present study. In contrast, Turkish coffee (987 mg GAE/100 mL) and American coffee (505 mg GAE/100 mL) showed lower TPC levels, aligning with the trend observed for brewing methods with gentler extraction processes, such as the French Press, V60, and Simple Infusion. This pattern is further supported by Caporaso et al. [37], who found the highest phenolic content in Espresso compared to American, Neapolitan, and Moka methods, and by Chavez et al. [38], who reported similar findings, with Espresso showing the highest TPC and comparable values for French Press and V6, which is consistent with the present study results.

It is worth noting that the TPC of the brews can vary depending on the coffee species. Robusta had a higher TPC than Arabica in all brewing methods, except for the V60 method. A study by Fărcaş et al. [39] also observed the same relationship, with Robusta coffee infusions showing higher TPC than Arabica. On the other hand, in the V60 method, compared to French Press and Simple Infusion, the continuous flow of water and finer grinding method promoted a more efficient release of chlorogenic acid derivative compounds, more polar and thermolabile, predominant in Arabica [40,41].

The darker roasting grade of coffee may lead to a decrease in phenolic compounds, as suggested by previous studies [40,41,42]. A similar trend was observed in the present study, although it was not statistically significant. The lack of apparent differences between roasting grades may be due to the balance between thermal degradation of phenolic compounds and the formation of new derivatives with reducing properties, such as melanoidins [43]. Moreover, the kinetics of thermal degradation should be considered, as different phenolic compounds decompose at varying rates, leading to complex changes in total phenolic content during roasting. In addition, changes in bean porosity and the structure of cell walls during roasting can facilitate the extraction process [42]. It is also worth noting that the method used to determine TPC (Folin–Ciocalteu reaction) is prone to interference and does not always accurately reflect the actual polyphenol content, which could have affected the final results. Interestingly, this study have reported contradictory findings, showing that roasting degree can have a stronger impact on coffee quality than the brewing method. In particular, cold brewing of lightly roasted beans was reported to produce coffee with improved physicochemical and sensory properties, suggesting that under certain conditions, roasting may play a more decisive role than extraction parameters [42].

Concerning geographic origin, growing conditions may be important [33], but their effect on TPC could have been reduced by more substantial effects due to species and extraction methods.

Each of the methods analyzed (Espresso, Simple Infusion, French Press, and V60) had a different extraction profile for each element, highlighting the complexity of minerals release processes from coffee and the dependence on extraction parameters such as temperature, water contact time, grind level, and raw material to water ratio [11,12].

The highest Ca concentrations were recorded in brews prepared by the V60 method, which may result from the long contact time between water and coffee and the large filtration surface area, which allows more efficient dissolution and transport of Ca ions into solution [11,44]. Water flow, as highlighted by Vandeponseele et al. [45] and Moroney et al. [46], promotes efficient extraction of solutes. In contrast, Stelmach et al. [47] found that higher temperature increases Ca extraction by 20%. However, it is worth considering the influence of the type of water; the presence of Ca in the water may limit its diffusion from the coffee into the brew [11]. The variety of water sources (tap, filtered, distilled) used in the study may explain the discrepancies in the concentrations of this element [11,47,48,49,50,51] and cause ambiguity in the study results. The lowest Ca concentrations were observed in the Simple Infusion method, which may be due to the lack of water flow and limited contact between coffee and solvent [52].

The K content of coffee infusions significantly depends on the brewing method. As an easily soluble element, K is one of the most rapidly extracted compounds in coffee [53]. In our study, the highest concentrations were recorded in Espresso, possibly due to the high pressure and high coffee-to-water ratio, leading to a highly concentrated brew as indicated by Olechno et al. [11]. In the V60 method, the K content was more than twice as low despite the more prolonged contact between water and coffee. In contrast, Simple Infusion and French Press recorded the lowest K concentrations, possibly due to the less dynamic extraction process [11].

K, P, and Mg contents were also significantly higher in infusions prepared by the Espresso method, which could be attributed to the intense extraction conditions. However, it is important to note that the extraction process is influenced not only by temperature and pressure, but also by mass transfer kinetics, particle size distribution, and thermodynamic equilibria. Classical kinetic studies on caffeine extraction in coffee [54,55] showed that diffusion through the porous structure of the beans is rate-limiting; similar mechanisms likely affect the release of minerals and other bioactive compounds and the extraction process is further modulated by particle swelling, countercurrent water flow, and interactions with other soluble compounds [55]. The rate at which minerals are released depends on diffusion through the porous structure of coffee particles, the degree of particle fragmentation, and interactions with other soluble compounds, which may limit or enhance solubility. Differences in mineral content between brewing methods, as reported by Janda et al. [48] (with the highest Mg concentration was recorded in the Aeropress infusion, followed by Simple Infusion, Drip, and then Espresso) could therefore result from a combination of species, degree of grinding, the time of contact with water and the dynamic balance between solute solubility and extraction kinetics. These factors highlight the complexity of mineral extraction and the need to consider multiple mechanistic aspects when interpreting differences among brewing methods.

The highest Na concentration was recorded in the brew prepared using the V60 method. This can be attributed to the longer flow time of the water through the coffee and the use of a paper filter, which promotes efficient extraction. These results are consistent with the observations of Özdestan [49], who noted high Na content in the Turkish method, with a favorable ratio of extraction area to water volume. Although the Espresso method is characterized by intensive mechanical conditions, the shorter contact time between coffee and water (about 25–30 s) may limit Na extraction. Nevertheless, fine grinding and high pressure partially compensate for this effect, as confirmed by the results of Janda et al. [48], where using a large amount of finely ground coffee yielded high Na content even in the absence of pressure. The lowest Na concentration was recorded in Simple Infusion. Despite the long extraction time, the lack of liquid movement and medium grinding may have limited the efficiency of the process. Similar findings were observed by Gogoaşă et al. [50], who pointed out that brewing time alone does not guarantee effective extraction. Dynamic factors such as water flow or mechanical agitation may be key, which explains the higher Na concentrations obtained in our study in the French Press method and the study by Ashu and Chandravanshi [51], despite the shorter brewing time.

Both micronutrients Fe and Zn were present in the infusions in low concentrations, which may be explained by their limited solubility in water and tendency to form organic complexes that are difficult to dissolve [56]. Additional losses may have resulted from adsorption of ions on paper and metal filters. In the case of Zn, there were no significant differences between steaming methods, indicating its limited extraction regardless of extraction parameters. Discrepancies in literature data [48,51,57] suggest that factors such as coffee to water ratio or water type may affect Zn concentration in brews, although they are not decisive. For example, da Silva et al. [57] obtained higher Zn concentrations with more coffee, while Janda et al. [48] obtained lower Zn concentrations, despite the high coffee-to-water ratio. The influence of water type was confirmed by the results of Stelmach et al. [47], which showed that tap water favored higher Zn content than mineral water. For Fe, only the V60 method differed significantly from the others. This may be due to milder extraction and stabilization conditions of Fe^2+^ with fewer phenolic compounds [12].

The results indicated that the species of coffee (Arabica, Robusta, Blend) affects the content of some minerals in the brews to different degrees, especially Mg, Zn, and Fe. These differences may be due to genetics, soil composition, cultivation, and processing techniques. According to the literature, Arabica and Robusta can differ in their mineral content, although the differences are not always significant [58]. In contrast, according to Dippong et al. [59], Robusta grains contain more Na, K, Ca, Mg, Fe, Cu, Mn, Zn, P, and polyphenols than Arabica. In the present study, Mg content was higher in Arabica brews regardless of brewing method. This may be due to the higher bioavailability of this element in the plant’s tissues or to the growing environment involving Mg-rich volcanic soils found at high altitudes [60].

Zn showed variation in content only in Espresso, Zn content was significantly lower in Arabica than in Robusta and Blend. Zn is among the minerals strongly dependent on soil conditions, substrate acidity, and competing ions (e.g., Fe, Cu). Differences may also result from processing technology, with Arabica more often subjected to wet fermentation, which can lead to Zn leaching from the grain surface. Stelmach et al. [47] also showed higher Zn concentrations in blends than in pure Arabica.

In the case of Fe, a significantly higher content was reported in V60 infusions from Robusta beans than from Arabica. According to Tagliaferro et al. [61], the higher Fe content may be related to the presence of soil residues on the grains, as the wet treatment used more often for Arabica removes them more effectively, which could explain the lower Fe content. However, previous studies [48,50] did not consider the origin of the coffee when analyzing Fe content.

In contrast, K, P, Na, and Ca content did not differ significantly between species, suggesting that their extraction depends primarily on brewing conditions. It is worth noting that many authors do not specify the species of coffee analyzed [11,12,44,51], which makes comparisons between studies and interpretation of mineral composition data difficult.

The temperature, pH, and ionic composition of the extraction medium are additional factors that can alter the extraction of minerals and bioactive compounds in coffee. Recent studies have shown that extraction temperature strongly influences the physicochemical parameters of coffee, including refractive index, pH, and total dissolved solids, with lower brewing temperatures resulting in higher pH values and generally lower extraction yields [62]. Similarly, the pH of the extraction medium plays a key role: an increase in pH from 7.0 to 9.0 resulted in a 24.5% decrease in total phenolic content in coffee extracts, with a significant reduction in chlorogenic acid and other phenolic compounds, highlighting the instability of these molecules under alkaline conditions [63]. Furthermore, the ionic composition of brewing water can influence the extraction efficiency of certain coffee compounds. For example, dissolved cations such as Mg^2+^ and Ca^2+^ in the water may interact with nucleophilic motifs in coffee compounds, enhancing the extraction of less soluble compounds, including chlorogenic acid lactones, while the effect on readily soluble organic acids appears to be negligible [64]. Finally, correlations between mineral content in different brews indicate that ionic interactions and matrix effects may shape the mineral profile of coffee brews in complex and method-dependent ways [48]. In summary, these results emphasize that when interpreting differences in the composition of minerals and bioactive compounds across different studies, it is important to consider not only the brewing method and coffee species, but also the extraction temperature, pH, and ionic strength of the medium.

At the beginning of the discussion, the strengths of this study were highlighted, but despite the efforts made, certain limitations should also be acknowledged. Two modern analytical methods were used in the study: HPLC to determine the caffeine content and ICP-OES to analyze the mineral composition. The total content of polyphenols was determined using the Folin–Ciocalteu method, which only allows the determination of the total amount of phenolic compounds without their detailed qualitative and quantitative characterization. It should also be noted that reducing sugars, ascorbic acid, and amino acids present in coffee may interfere with the Folin–Ciocalteu assay, potentially leading to false-positive readings. The results presented should therefore be considered as preliminary studies that can serve as a starting point for future, more in-depth analyses.

Another limitation could be the fact that the analyzed coffee samples may introduce potential bias as they were based solely on the manufacturers’ claims. On the one hand, this is a limitation; on the other hand, it reflects the real market conditions and products that consumers actually encounter.

The aforementioned factors may have influenced the results obtained. Nevertheless, this study has led to a deeper understanding of the changes in the chemical profile of coffee depending on the brewing method used, as well as on raw material and technological factors. Overall, the Espresso method generally showed higher contents of caffeine, TPC, K, Mg, and P, whereas the V60 method exhibited relatively higher Ca, Fe, and Na contents. French Press and Simple Infusion displayed intermediate values. Among the coffee species, Robusta tended to contain more caffeine and TPC than Arabica. Mg was a mineral that was found to be higher in Arabica coffee. The Blends had intermediate parameters reflecting the contribution of each variety to the chemical profile. In contrast, the degree of roasting and the geographical origin of the beans appeared to have limited influence on the chemical composition of the brews.

## 4. Materials and Methods

### 4.1. Materials

**Figure 9:**
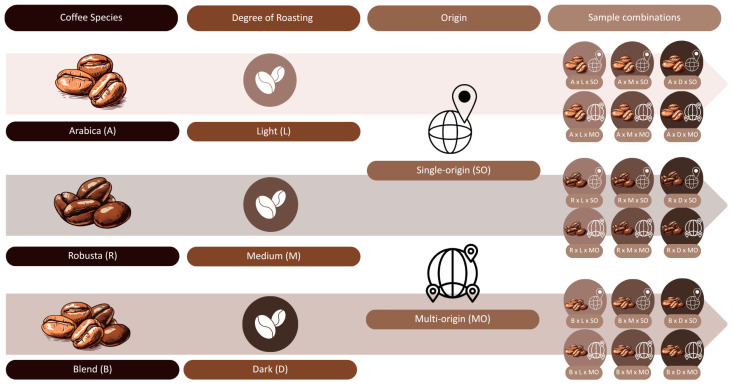
Coffee samples included in the study, categorized by species (Arabica, Robusta, Blend), roast degree (light, medium, dark), and geographical origin (single-origin, multi-origin). Canva (https://www.canva.com/) was used to generate the figure.

The study analyzed roasted coffee that was purchased from specialized online retailers providing detailed information on species, roast degree, and country of origin. These data served as the basis for sample classification and ensured a basic level of traceability and standardization. The study included 18 coffee samples selected based on three key factors: species, roast grade, and origin. Each sample came from one of the following combinations of parameters—coffee species: Robusta, Arabica, Blend; roast grade: light, medium, dark; origin: single-origin, multi-origin, as shown in Figure 9. The aim of the study was not to assess the commercial quality of the products, but to investigate the effect of brewing methods on the chemical composition of coffee infusions in a representative set of coffees available to consumers.

In this study, different coffee samples selected according to species, roast degree, and origin were considered as biological replicates. For each sample, three independent chromatographic analyses were performed, which were treated as technical replicates.

### 4.2. Brewing Methods

Four different brewing methods were used in the study, differing in terms of coffee grind, coffee to water ratio and brewing times, to assess the effects of these parameters on the physicochemical properties of the brewed beverages. Details of the individual methods are listed below:

Espresso: Very finely ground coffee (7 g), brewed with 25 mL of water at 90–96 °C. The brewing time was 25–30 s, and the ratio of coffee to water was 28:100.

Simple Infusion: Medium-ground coffee (15 g), brewed with 250 mL of water at 92 °C. The brewing time was 5 min. The ratio of coffee to water was 6:100.

French Press: Coarsely ground coffee (15 g), brewed with 250 mL of water at 93 °C. The brewing time was 5 min, after which the plunger was pressed to separate the coffee grounds from the brewed coffee. The ratio of coffee to water was 6:100.

V60: Medium-fine ground coffee (15 g), brewed with 250 mL 93 °C hot water for 3.5 min. The water was infused in two steps: initially, 70 mL of water was left for 30 s for initial brewing, then 180 mL of water was infused in concentric circles. The ratio of coffee to water was 6:100.

### 4.3. Assessment of Physicochemical Parameters

Before analyzing and evaluating selected parameters, all samples were brought to 20 °C to ensure uniform measurement conditions. The pH value of the coffee brew was measured using an Elmetron CP-105 pH meter, which was calibrated with three buffer solutions with pH values of 4.00, 7.00, and 9.00 before analysis. The Total Dissolved Solids (TDS) value was calculated based on the percentage extract (Brix) reading obtained with a refractometer (OPTi Digital, Bellingham + Stanley, Xylem, Nottingham, UK), according to the formula: TDS (%) = Brix (%) × 0.85. A factor of 0.85 was included, and a correction was adjusted for coffee solutions. The extraction efficiency was calculated as the ratio of the product of the TDS value and the mass of the brewed coffee to the mass of ground coffee used in the brewing process. Table 5 shows the physicochemical parameters of coffee infusions depending on the brewing method.

### 4.4. Determination of Caffeine Content

An Ultimate 3000 high-performance liquid chromatograph (Thermo Scientific, Waltham, MA, USA) was used to determine caffeine concentrations. The infusions, prepared according to the accepted extraction methods, were cooled to room temperature. The samples were then diluted and filtered using a PTFE membrane filter with a pore diameter of 0.22 μm. The analysis used a BDS C18 HYPERSIL reversed-phase column (250 mm × 4.6 mm, 5 µm, Thermo Scientific, USA). The mobile phase consisted of a mixture of ultrapure water obtained from a HLP Smart demineralizer (Hydrolab, Straszyn, Poland) and methanol (J.T.Baker, Avantor Performance Materials Poland SA, Gliwice, Poland) in a volume ratio of 70:30. The flow rate of the mobile phase was 1 mL/min, the volume of the injected sample was 10 µL, and the UV detector was set at a wavelength of 272 nm. The column was thermostated at 25 °C. Each sample was analyzed in triplicate, with a total run time of 8 min per analysis. The retention time for caffeine was 5.7 min. Representative HPLC chromatograms of the caffeine standard and coffee samples prepared by the four brewing methods are provided in the Supplementary Materials (Figure S1).

Caffeine standard solutions (Supelco, Merck KGaA, Darmstadt, Germany) with concentrations ranging from 5 to 1000 mg/L were used to prepare the calibration curve. A ten-point calibration plot was developed based on the data obtained, for which the coefficient of determination was R^2^ = 0.99953. The results of the analysis were calculated from the calibration curve. Limits of detection (LOD) and quantification (LOQ) were calculated based on signal-to-noise ratios of 3 and 10, respectively, yielding typical values of 0.043 mg/L and 0.144 mg/L for caffeine. Method precision, evaluated as the relative standard deviation (%RSD), was 1.46%, demonstrating high repeatability and reliability of the analytical procedure.

### 4.5. Determination of TPC

With some modifications, the TPC value in coffee samples was determined using the Folin–Ciocalteu method [17]. To 400 µL of diluted coffee brew (50 µL of coffee extracts diluted with distilled water to 2.5 mL), 200 µL of Folin–Ciocalteu reagent and 600 µL of 20% sodium carbonate solution were added, and the total was diluted to a volume of 4 mL. The samples were incubated in the dark for 120 min, after which the absorbance at 765 nm was measured. Results were expressed in milligrams of gallic acid equivalent (GAE) per 100 mL coffee.

### 4.6. Determination of Mineral Content

The coffee brew’s minerals were analyzed using an Inductively Coupled Plasma–Optical Emission Spectrometry (ICP-OES) spectrophotometer (Spectroblue, AMETEK Inc., Meerbusch, Germany). To prepare the samples, 0.75 mL of coffee infusion was taken into teflon vessels, and then 4 mL of 65% nitric acid (HNO_3_) was added, leaving the samples for 30 min. Then, 1 mL of 30% hydrogen peroxide (H_2_O_2_) solution was added for oxidation and further mineralization. The samples were mineralized in a microwave system (Anton Paar, Graz, Austria) using the program: 35 min at 180 °C, with the temperature rising to 180 °C for 15 min, and then maintaining this temperature for 20 min. After digestion, samples were transferred to test tubes and diluted 5 times. The samples were stored in the refrigerator at 4 °C until analysis. The content of macronutrients: Ca, K, Mg, Na, P, and micronutrients: Fe, Zn were evaluated.

### 4.7. Statistical Analysis

Principal Component Analysis (PCA) and Partial Least Squares–Discriminant Analysis (PLS-DA) were carried out to assess the potential separation of samples by brewing method, species, roasting grade, and coffee bean origin, and to identify the variables most influential in differentiating the groups analyzed. PCA and PLS-DA were conducted using the MetaboAnalyst online platform. Prior to multivariate analyses (PCA and PLS-DA) performed in MetaboAnalyst, the data were subjected to preprocessing, including sample normalization using the sum method, data transformation via square root of the values, and Pareto scaling to ensure appropriate weighting of features and reliable interpretation of the multivariate results. Other statistical analyses were performed using the Statistica software version 13.0 (StatSoft Inc., Tulsa, OK, USA). For comparisons between two groups, either Student’s *t*-test or the non-parametric Mann–Whitney U test was applied, depending on whether the data conformed to a normal distribution. For comparisons between more than two groups, analysis of variance with Tukey’s post hoc test or the nonparametric Kruskal–Wallis test was used, depending on whether the test assumptions were met. Results with a significance level of *p* < 0.05 were considered statistically significant.

## 5. Conclusions

The analysis showed that both the brewing method (Espresso, Simple Infusion, French Press, and V60) and the coffee species (Arabica, Robusta, and Blend) tended to influence the chemical profile of the resulting brews. The results of the present study suggest that the choice of brewing method and coffee species can influence the caffeine, antioxidant, and mineral content of the brew. Consumers who prefer an intense flavor and a high caffeine content should choose Robusta coffee or blends containing Robusta that are brewed with Espresso. On the other hand, Arabica coffee brewed with V60 may be a better choice for consumers concerned about antioxidant intake due to its high TPC. Although a single serving of coffee does not cover a significant portion of daily mineral requirements, regular consumption of infused beverages can provide Fe, Ca, Mg or K, especially Arabica coffee prepared with V60. These findings may have practical applications both for consumers, who can consciously choose coffee according to their preferences and nutritional needs, and for the coffee industry to optimize the preparation of infusions. Further research, including expanded brewing parameters and sensory analysis, may contribute to a better understanding of the relationship between coffee’s chemical composition and organoleptic properties.

## Figures and Tables

**Table 1:** Minerals Composition of Brewing Method.

Parameter [mg/100 mL]	Espresso	Simple Infusion	French Press	V60	*p* Value
Ca	4.35 ± 0.48 b	3.76 ± 0.73 a	4.22 ± 0.67 ab	6.53 ± 0.75 c	<0.001
Fe	0.16 ± 0.02 a	0.15 ± 0.05 a	0.16 ± 0.03 a	0.27 ± 0.05 b	<0.001 *
K	37.63 ± 3.44 d	15.43 ± 2.91 a	18.53 ± 2.25 ab	21.62 ± 1.27 bc	<0.001 *
Mg	4.21 ± 0.55 c	2.24 ± 0.51 a	2.27 ± 0.42 a	2.83 ± 0.41 b	<0.001
Na	4.48 ± 0.54 b	3.60 ± 0.73 a	4.34 ± 0.43 b	5.97 ± 0.63 c	<0.001 *
P	5.67 ± 0.54 c	3.43 ± 0.65 a	3.67 ± 0.30 ab	4.18 ± 0.35 b	<0.001 *
Zn	0.04 ± 0.01 a	0.06 ± 0.03 a	0.04 ± 0.01 a	0.04 ± 0.01 a	0.0604 *

Abbreviations: *—denotes the application of the Kruskal–Wallis test. Values are presented as mean ± standard deviation (*n* = 3). Different lowercase letters denote statistically significant differences between brewing methods (*p* < 0.05), as determined by one-way ANOVA followed by Tukey’s post hoc test or, when normality assumptions were not met, by the non-parametric Kruskal–Wallis test.

**Table 2:** Minerals Compositions of Coffee Species.

	Parameter [mg/100 mL]	Arabica	Blend	Robusta	*p* Value
Espresso	Ca	4.65 ± 0.50 a	4.16 ± 0.31 a	4.24 ± 0.53 a	0.1653
	Fe	0.15 ± 0.03 a	0.15 ± 0.01 a	0.17 ± 0.03 a	0.4199
	K	37.89 ± 2.55 a	36.98 ± 1.63 a	38.10 ± 5.92 a	0.8590
	Mg	4.79 ± 0.34 b	4.03 ± 0.29 a	3.82 ± 0.44 a	0.0008
	Na	4.43 ± 0.60 a	4.54 ± 0.59 a	4.45 ± 0.53 a	0.8304 *
	P	5.56 ± 0.51 a	5.53 ± 0.41 a	5.92 ± 0.66 a	0.4004
	Zn	0.04 ± 0.01 b	0.03 ± 0.00 a	0.04 ± 0.01 b	0.0031 *
Simple infusion	Ca	4.22 ± 0.48 a	3.63 ± 0.54 a	3.43 ± 0.94 a	0.1427 *
	Fe	0.16 ± 0.07 a	0.13 ± 0.05 a	0.17 ± 0.05 a	0.4049 *
	K	16.32 ± 2.38 a	16.47 ± 2.06 a	13.51 ± 3.48 a	0.1372
	Mg	2.61 ± 0.37 a	2.06 ± 0.38 a	2.05 ± 0.61 a	0.0907
	Na	3.81 ± 0.32 a	3.78 ± 0.82 a	3.23 ± 0.87 a	0.3137
	P	3.47 ± 0.45 a	3.24 ± 0.38 a	3.55 ± 0.99 a	0.7441
	Zn	0.07 ± 0.05 a	0.05 ± 0.02 a	0.05 ± 0.02 a	0.5539 *
French Press	Ca	4.51 ± 0.64 a	3.95 ± 0.25 a	4.21 ± 0.93 a	0.3734
	Fe	0.17 ± 0.03 a	0.15 ± 0.02 a	0.16 ± 0.03 a	0.3310
	K	19.21 ± 0.91 a	17.72 ± 1.91 a	18.67 ± 3.37 a	0.2621 *
	Mg	2.76 ± 0.24 b	2.03 ± 0.09 a	2.01 ± 0.33 a	0.0001
	Na	4.54 ± 0.50 a	4.23 ± 0.33 a	4.22 ± 0.43 a	0.3802
	P	3.75 ± 0.18 a	3.56 ± 0.26 a	3.70 ± 0.42 a	0.3857 *
	Zn	0.04 ± 0.02 a	0.04 ± 0.01 a	0.04 ± 0.01 a	0.9011
V60	Ca	6.74 ± 0.71 a	6.10 ± 0.94 a	6.74 ± 0.47 a	0.2469
	Fe	0.28 ± 0.06 ab	0.23 ± 0.02 a	0.29 ± 0.02 b	0.0159 *
	K	21.90 ± 1.34	21.30 ± 1.17	21.65 ± 1.44	0.6300 *
	Mg	3.24 ± 0.30 b	2.60 ± 0.32 a	2.67 ± 0.31 a	0.0049
	Na	6.21 ± 0.70 a	5.42 ± 0.18 a	6.29 ± 0.51 a	0.0519 *
	P	4.30 ± 0.42 a	4.12 ± 0.23 a	4.12 ± 0.38 a	0.5808
	Zn	0.04 ± 0.01 a	0.03 ± 0.01 a	0.04 ± 0.01 a	0.1794

Abbreviations: *—denotes the application of the Kruskal–Wallis test. Values are presented as mean ± standard deviation (*n* = 3). Different lowercase letters denote statistically significant differences between brewing methods (*p* < 0.05), as determined by one-way ANOVA followed by Tukey’s post hoc test or, when normality assumptions were not met, by the non-parametric Kruskal–Wallis test.

**Table 3:** The content of caffeine, phenolic compounds, and minerals in one serving of coffee, depending on the brewing method (Espresso—25 mL; other—250 mL).

Parameter (mg per Serving)	Espresso	Simple Infusion	French Press	V60
Caffeine	63.06	195.90	206.88	185.48
TPC	664.08	2948.13	2421.60	2396.93
Ca	1.09	9.40	10.55	16.33
Mg	1.05	5.60	5.68	7.08
K	9.41	38.58	46.33	54.05
Na	1.12	9.00	10.85	14.93
P	1.42	8.58	9.18	10.45
Fe	0.04	0.38	0.40	0.68
Zn	0.01	0.15	0.10	0.10

Abbreviations: TPC—total phenolic content.

**Table 4:** The contribution of one serving of coffee to the daily requirement for caffeine and minerals, depending on the brewing method (Espresso—25 mL; other—250 mL).

Parameter (mg per Serving)	EFSA Recommendation	Espresso [% RDI]	Simple Infusion [% RDI]	French Press [% RDI]	V60 [% RDI]
Caffeine	400	15.77	48.98	51.72	46.37
Ca	1000 (18–24 years); 950 (≥25 years)	0.11 *	0.99 *	1.11 *	1.72 *
Mg	M: 350 F: 300	0.30 0.35	1.60 1.87	1.62 1.89	2.02 2.36
K	3500	0.27	1.10	1.32	1.54
Na	2000	0.06	0.45	0.54	0.75
P	550	0.26	1.56	1.67	1.90
Fe	M: 11 F: 16 (premenopausal); 11 (postmenopausal)	M: 0.36 F: 0.25	M: 3.45 F: 2.38	M: 3.64 F: 2.50	M: 6.18 F: 4.25
Zn	M: 9.4–16.3 F: 7.5–12.7	M: 0.11 F: 0.13	M: 1.60 F: 2.00	M: 1.06 F: 1.33	M: 1.06 F: 1.33

Abbreviations: *—based on the lower reference value of the recommended intake; F—female; M—male; RDI—Recommended Daily Intake.

**Table 5:** Physicochemical characteristics of coffee brews by brewing method.

Brewing Methods	Espresso	Simple Infusion	French Press	V60
pH	5.48 ± 0.22	5.84 ± 0.22	5.84 ± 0.23	5.78 ± 0.26
TDS (%)	3.65 ± 0.48	1.40 ± 0.13	1.35 ± 0.24	1.18 ± 0.10
Extraction yield (%)	11.65 ± 1.49	18.97 ± 1.78	18.88 ± 2.83	16.11 ± 1.26
Brew Mass (g)	22.43 ± 1.43	192.11 ± 4.69	221.61 ± 2.52	204.26 ± 2.99
Brew volume (mL)	24.47 ± 1.42	204.10 ± 5.32	214.93 ± 2.43	208.17 ± 3.5

Abbreviations: TDS—Total Dissolved Solids.

## Data Availability

The original contributions presented in this study are included in the article/Supplementary Material. Further inquiries can be directed to the corresponding author.

## Supplementary Materials

The following supporting information can be downloaded at: https://www.mdpi.com/article/10.3390/molecules30204080/s1, Table S1: Caffeine content, total phenols and minerals within degree of roasting; Table S2: Caffeine content, total phenols and minerals within origin; Figure S1: Representative HPLC chromatograms: (a) standard solution of caffeine; (b) coffee prepared by the espresso method; (c) coffee prepared by the simple infusion method; (d) coffee prepared using a French press; and (e) coffee prepared using the V60 method.
