# Supplementary material for: Influence of Brewing Methods on the Bioactive and Mineral Composition of Coffee Beverages
Source: Molecules. 2025 Oct 14;30(20):4080. doi: 10.3390/molecules30204080 (PMC12565998; doi:10.3390/molecules30204080)
Supplement: Supplementary file 1 [file molecules-30-04080-s001.zip › molecules-3905510-supplementary.pdf]

Table S1. Caffeine content, total phenols and minerals within degree of roasting.

Brewing method	Compound	Light	Medium	Dark	P value
Espresso	Caffeine [mg/100 ml]	227.67 ± 49.21a	249.60 ± 63.07a	279.49 ± 84.17a	0.4262
	TPC [mg GAE/100ml]	2652.10 ± 360.79a	2687.74 ± 330.46a	2629.14 ± 161.32a	0.9428
	Ca [mg/100ml]	4.48 ± 0.43a	4.53 ± 0.58a	4.04 ± 0.31a	0.1501
	Fe [mg/100ml]	0.16 ± 0.03a	0.17 ± 0.03a	0.15 ± 0.02a	0.3577*
	K [mg/100ml]	36.80 ± 4.90a	37.98 ± 2.86a	38.21 ± 2.36a	0.7811
	Mg [mg/100ml]	4.21 ± 0.54a	4.30 ± 0.56a	4.13 ± 0.62a	0.8377*
	Na [mg/100ml]	4.38 ± 0.46a	4.74 ± 0.68a	4.30 ± 0.45a	0.3675*
	P [mg/100ml]	5.66 ± 0.56a	5.86 ± 0.56a	5.49 ± 0.51a	0.5149
	Zn [mg/100ml]	0.04 ± 0.01a	0.04 ± 0.01a	0.04 ± 0.01a	0.9154
Simple Infusion	Caffeine [mg/100 ml]	86.64 ± 35.68a	78.30 ± 27.56a	70.15 ± 35.58a	0.6961
	TPC [mg GAE/100ml]	1318.38 ± 309.78a	1166.76 ± 213.70a	1052.60 ± 114.73a	0.4594*
	Ca [mg/100ml]	4.15 ± 0.69a	3.50 ± 0.77a	3.65 ± 0.68a	0.1720*
	Fe [mg/100ml]	0.15 ± 0.04a	0.14 ± 0.04a	0.17 ± 0.07a	0.5220
	K [mg/100ml]	16.74 ± 1.77a	13.17 ± 3.55a	16.39 ± 1.92a	0.0533
	Mg [mg/100ml]	2.50 ± 0.32a	2.14 ± 0.46a	2.08 ± 0.68a	0.3314
	Na [mg/100ml]	3.92 ± 0.82a	3.26 ± 0.82a	3.63 ± 0.42a	0.5260*
	P [mg/100ml]	3.81 ± 0.33a	3.36 ± 0.84a	3.18 ± 0.57a	0.2686
	Zn [mg/100ml]	0.08 ± 0.05a	0.04 ± 0.01a	0.04 ± 0.02a	0.1076*
French Press	Caffeine [mg/100 ml]	76.98 ± 17.64a	88.66 ± 23.95a	82.62 ± 22.60a	0.5225*
	TPC [mg GAE/100ml]	995.22 ± 270.77a	986.16 ± 146.98a	924.54 ± 189.47a	0.5713*
	Ca [mg/100ml]	4.09 ± 0.66a	4.49 ± 0.71a	4.10 ± 0.67a	0.3679*
	Fe [mg/100ml]	0.14 ± 0.02a	0.17 ± 0.03a	0.16 ± 0.03a	0.1231
	K [mg/100ml]	16.31 ± 2.29a	19.38 ± 1.64bc	19.91 ± 0.47c	**0.0037**
	Mg [mg/100ml]	2.15 ± 0.47a	2.36 ± 0.35a	2.28 ± 0.49a	0.7165
	Na [mg/100ml]	4.02 ± 0.29a	4.49 ± 0.30a	4.53 ± 0.49a	0.0698
	P [mg/100ml]	3.53 ± 0.41a	3.76 ± 0.29a	3.72 ± 0.09a	0.5668*
	Zn [mg/100ml]	0.04 ± 0.01a	0.03 ± 0.00a	0.04 ± 0.02a	0.9769*

V60	Caffeine [mg/100 ml]	74.78 ± 21.01a	73.95 ± 13.15a	73.83 ± 20.78a	0.9954
	TPC [mg GAE/100ml]	1102.13 ± 328.71a	893.13 ± 75.06a	880.51 ± 196.21a	0.4594*
	Ca [mg/100ml]	6.67 ± 0.79a	6.46 ± 0.74a	6.46 ± 0.84a	0.8677
	Fe [mg/100ml]	0.27 ± 0.06a	0.25 ± 0.04a	0.27 ± 0.04a	0.7616
	K [mg/100ml]	21.95 ± 0.61a	20.76 ± 1.87a	22.14 ± 0.53a	0.1641*
	Mg [mg/100ml]	2.97 ± 0.29a	2.76 ± 0.46a	2.77 ± 0.50a	0.6275
	Na [mg/100ml]	5.89 ± 0.62a	5.74 ± 0.43a	6.29 ± 0.76a	0.3087*
	P [mg/100ml]	4.30 ± 0.21a	4.11 ± 0.40a	4.13 ± 0.42a	0.6222
	Zn [mg/100ml]	0.05 ± 0.01b	0.04 ± 0.01ab	0.03 ± 0.01a	**0.0182**

Abbreviations: * - denotes the application of the Kruskal–Wallis test.

Table S2. Caffeine content, total phenols and minerals within origin.

Brewing method	Compound	Single-origin	Multi-origin	p-value
Espresso	Caffeine [mg/100 ml]	250.35 ± 67.75a	254.15 ± 69.60a	0.9082
	TPC [mg GAE/100ml]	2649.08 ± 294.17a	2663.57 ± 283.77a	0.9166
	Ca [mg/100ml]	4.47 ± 0.58a	4.23 ± 0.36a	0.2896
	Fe [mg/100ml]	0.17 ± 0.03a	0.15 ± 0.02a	0.1772
	K [mg/100ml]	37.48 ± 2.68a	37.76 ± 4.17a	0.8742
	Mg [mg/100ml]	4.21 ± 0.44a	4.22 ± 0.66a	0.9759
	Na [mg/100ml]	4.62 ± 0.53a	4.33 ± 0.55a	0.1223*
	P [mg/100ml]	5.71 ± 0.57a	5.63 ± 0.53a	0.7537
	Zn [mg/100ml]	0.04 ± 0.01a	0.03 ± 0.01a	0.3720
Simple Infusion	Caffeine [mg/100 ml]	75.55 ± 33.10a	81.17 ± 32.40a	0.7209
	TPC [mg GAE/100ml]	1159.92 ± 282.37a	1198.58 ± 206.96a	0.7447
	Ca [mg/100ml]	3.43 ± 0.64a	4.10 ± 0.68b	**0.0469**
	Fe [mg/100ml]	0.15 ± 0.05a	0.16 ± 0.06a	0.5664
	K [mg/100ml]	14.21 ± 3.34a	16.66 ± 1.85a	0.0720
	Mg [mg/100ml]	2.18 ± 0.43a	2.30 ± 0.61a	0.6301
	Na [mg/100ml]	3.21 ± 0.63a	3.99 ± 0.62b	**0.0104***
	P [mg/100ml]	3.44 ± 0.69a	3.42 ± 0.64a	0.9568

	Zn [mg/100ml]	0.05 ± 0.02a	0.06 ± 0.05a	0.8852*
French Press	Caffeine [mg/100 ml]	82.90 ± 21.56a	82.60 ± 21.42a	0.9769
	TPC [mg GAE/100ml]	997.23 ± 224.64a	940.05 ± 177.98a	0.5078*
	Ca [mg/100ml]	4.10 ± 0.57a	4.35 ± 0.76a	0.4349
	Fe [mg/100ml]	0.16 ± 0.03a	0.16 ± 0.03a	0.7573*
	K [mg/100ml]	18.53 ± 1.91a	18.54 ± 2.66a	0.9947
	Mg [mg/100ml]	2.21 ± 0.36a	2.32 ± 0.49a	0.5950
	Na [mg/100ml]	4.36 ± 0.51a	4.31 ± 0.35a	0.8113
	P [mg/100ml]	3.64 ± 0.29a	3.71 ± 0.32a	1.0000*
	Zn [mg/100ml]	0.04 ± 0.02a	0.04 ± 0.01a	0.6287
V60	Caffeine [mg/100 ml]	76.35 ± 18.90a	72.02 ± 16.93a	0.6163
	TPC [mg GAE/100ml]	906.22 ± 233.60a	1011.32 ± 239.81a	0.1853*
	Ca [mg/100ml]	6.45 ± 0.73a	6.61 ± 0.80a	0.6581
	Fe [mg/100ml]	0.25 ± 0.04a	0.28 ± 0.05a	0.2652
	K [mg/100ml]	21.48 ± 1.39a	21.75 ± 1.20a	0.6658
	Mg [mg/100ml]	2.78 ± 0.35a	2.89 ± 0.48a	0.5703
	Na [mg/100ml]	5.80 ± 0.59a	6.14 ± 0.65a	0.2605
	P [mg/100ml]	4.21 ± 0.40a	4.15 ± 0.30a	0.7442
	Zn [mg/100ml]	0.04 ± 0.01a	0.04 ± 0.01a	0.9852

Abbreviations: *- denotes the application of the Mann-Whitney U test.

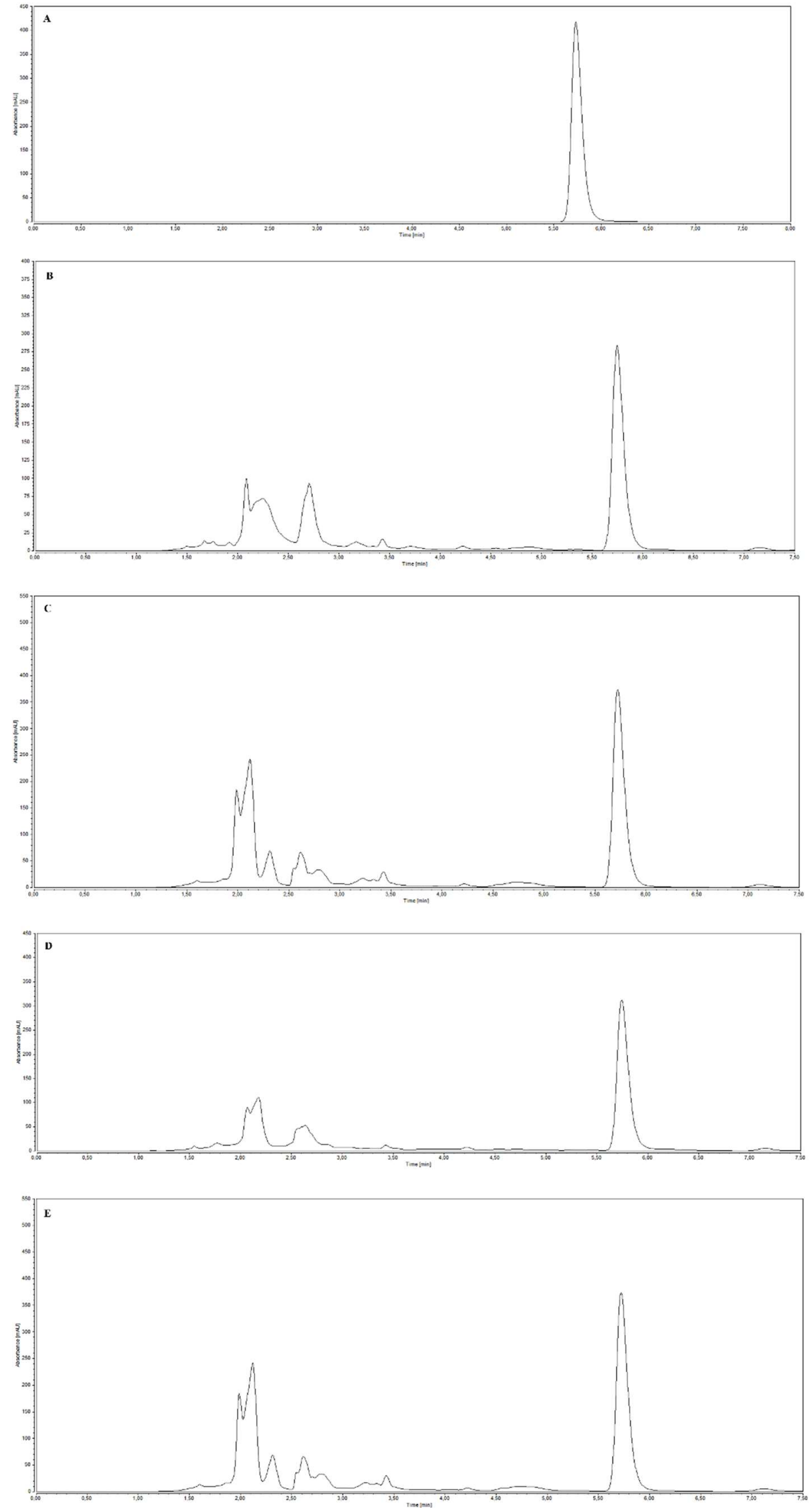

Figure S1. Representative HPLC chromatograms: (a) standard solution of caffeine; (b) coffee prepared by the espresso method; (c) coffee prepared by the simple infusion method; (d) coffee prepared using a French press; and (e) coffee prepared using the V60 method.
